# Assessment of stress and associated factors in employees of a public higher education institution

**DOI:** 10.47626/1679-4435-2022-700

**Published:** 2023-02-03

**Authors:** Nathália Teixeira Fonseca, Alessandra Silva Freire, Roberta Mendes Abreu Silva, Luís Rogério Cosme Silva Santos, Daniela da Silva Rocha, Márcio Vasconcelos Oliveira, Vanessa Moraes Bezerra

**Affiliations:** 1 Nutrition, Universidade Federal da Bahia (UFBA), Vitória da Conquista, BA, Brazil; 2 Collective Health, UFBA, Vitória da Conquista, BA, Brazil

**Keywords:** stress, occupational health, epidemiological studies, estresse, saúde do trabalhador, estudos epidemiológicos

## Abstract

**Introduction:**

Occupational stress is considered as the negative imbalance between work demands and resources, and it can generate consequences to an individual’s health and interfere with his or her quality of life.

**Objectives:**

To investigate stress and its associated factors among employees of a higher education institution through a cross-sectional study (at the baseline of a longitudinal study) including 176 individuals aged 18 years or older. Sociodemographic characteristics related to physical surroundings, lifestyle, working conditions, and health and illness were tested as explanatory variables.

**Methods:**

Stress was estimated using prevalence rate, prevalence ratio (PR), and a 95% confidence interval. For a multivariate analysis, we employed a Poisson regression model with robust variance, where a p-value ≤ 0.05 was considered significant.

**Results:**

The prevalence of stress was 22.7% (16.48-28.98). This study noticed that depressive individuals, professors, and those who self-assessed their health as poor or very poor had a positive association with stress within the studied population.

**Conclusions:**

Studies of this type are important for identifying characteristics in this population that could contribute to the planning of public policies in order to improve the quality of life of employees of public institutions.

## Abstract (pt)

**Introdução:**

O estresse ocupacional é considerado como o desequilíbrio negativo entre demandas de emprego e recursos de trabalho, podendo gerar consequências à saúde e interferir na qualidade de vida dos indivíduos.

**Objetivos:**

Investigar o estresse e os fatores associados em funcionários de uma instituição de ensino superior, através de um estudo transversal (linha de base de um estudo longitudinal) composto por 176 indivíduos de 18 anos ou mais. Variáveis sociodemográficas relacionadas ao entorno físico, estilo de vida, condições de trabalho e saúde e doença foram testadas como explicativas.

**Métodos:**

Foi realizada uma estimativa do estresse através da prevalência, razão de prevalência e intervalo de confiança de 95%. Para a análise multivariada, foi adotada a regressão de Poisson com variância robusta, sendo o valor de p ≤ 0,05 adotado como significativo.

**Resultados:**

A prevalência de estresse foi de 22,7% (16,48-28,98). O trabalho constatou que indivíduos depressivos, do grupo de docentes e aqueles que autoavaliaram sua saúde como ruim ou muito ruim apresentaram associação positiva com o estresse na população estudada.

**Conclusões:**

Pesquisas como esta são importantes para identificar características nessa população que podem contribuir com o planejamento de políticas públicas para melhorar a qualidade de vida dos funcionários da instituição pública.

## INTRODUCTION

In the last decades, the technological innovations and fast transformations that took place in work environments contributed to significant changes in the profile of work, which started requiring important abilities of physical, mental, and social adaptation; these, many times, end up frequently exposing the working population to situations involving stress, anxiety, distress, and emotional destabilization.^1,2^ Due to this paradoxical situation between positive and negative aspects, the stress related to the work environment has been thoroughly studied in the sense of identifying its participation in the etiology of worker illness.^3^

The word “stress” derives from Latin, being popularly employed in the 17th century with the meaning of “fatigue” and “tiredness.” According to Selye (1936), stress is a syndrome characterized by an ensemble of reactions developed by the body when subjected to a conflicting situation.^4^ Therefore, everything that causes a disruption of internal homeostasis, that is, requires some adaptation from the individual, can be called a stressor.^5^ In this sense, stress can be caused not only by psychological or psychic disturbances, but also by a set of daily events (external events) that can lead to psychological distress.^6^

Stress is a condition that can affect people of all ages. However, some population subgroups seem to be more vulnerable, such as women, older adults, people at higher vulnerability, individuals with a low education level, and workers.^7^ In this context, stress is considered a social and public health problem in the 21st century, being regarded as negative because it leads to damages and social issues such as mood changes, reduced productivity, cognitive and psychomotor alterations, and loss of initiative, among others.^8,9^ The intensity of the experienced stress is related to stressor severity and to the individual’s psychosocial coping resources. The World Health Organization (WHO) states that the stress load at the workplace and mental disorders within these places indicate a need to promote healthy work environments where physical health, safety, and wellbeing are achieved.^10^

Global health authorities recognize stress as the world’s largest epidemic of this century. In 2019, Gallup’s Global Emotions Report disclosed a study performed in 142 countries where one in every three interviewees experienced a lot of worry or stress, and at least one in every five people reported feeling sad or angry.^3^

The consequences of stress in the work domain are extensive, including depression, lack of initiative, lack of commitment, lack of motivation, irritability, impatience, difficulties with interpersonal relationships, loss of productivity, absenteeism and frequent tardiness, excess visits to the medical department, and medication dependence.^5^ Occupational stress is normally considered as the negative imbalance between work demands and resources experienced by workers and it can be generated by precarious working conditions, compromising an individual’s mental and physical health.^11^ A study by Cacciari et al indicates that chronic stress is an independent risk factor for the development of cardiovascular diseases, especially stroke.^12^

Within the context of occupational health problems, stress is seen as a worrisome aspect due to its serious consequences that can interfere with people’s quality of life. Therefore, this study proposes to assess stress levels among workers of a public higher education institution, as well as associated factors.

## METHODS

### STUDY DESIGN

This is a cross-sectional study (at the baseline of a longitudinal study) that aimed to investigate stress and its associated factors in a public higher education institution located in the southeast region of the state of Bahia (BA); the study was performed between January and June 2016.

### STUDY POPULATION

Out of a total of 204 employees (including professors, technicians, and outsourced employees), 191 individuals met the eligibility criteria. The study was performed at a public higher institution in Vitória da Conquista, BA. The following eligibility criteria were adopted: individuals aged 18 years or older, who were not on leave at the moment, and who consented to participate in a longitudinal follow-up by signing an informed consent form.

### PILOT STUDY

For verifying recruitment dynamics, testing the data collection instruments, and confirming the viability of our investigation, we performed a pilot study with employees of another university, who were comparable to the sample of this study and whose number comprised 8% of the final study population.

### DATA COLLECTION

Data were collected through individual in-person interviews, using a portable computer (HP Pocket Rx5710) for recording and storing data. The instrument adopted for the interviews was the National Health Survey (Pesquisa Nacional de Saúde [PNS]) semi-structured questionnaire, adapted to the study population. All interviewers were undergraduate students at the institution; they were organized in teams that were duly trained for data collection.

### SEMI-STRUCTURED INTERVIEWS

Individual interviews were performed using portable computers. The collected data were transferred to a dedicated database.

The instrument adopted for the interviews was based on instruments from national and international studies, such as: the questionnaire developed by the Belo Horizonte Observatory for Urban Health ([OSUBH], state of Minas Gerais [MG]) in the Move-se Hemominas project, performed with employees of the Belo Horizonte Blood Bank (Hemocentro) and its regional departments in Betim, Divinópolis, and Sete Lagoas (MG); the PNS questionnare^13^; the Surveillance of Risk and Protective Factors for Chronic Diseases by Telephone survey (Vigilância de Fatores de Risco e Proteção para Doenças Crônicas por Inquérito Telefônico [Vigitel])^14^; the International Physical Activity Questionnaire (IPAQ), long version; the World Health Organization Quality of Life instrument-Abbreviated version (WHOQOL-Bref) for quality of life; and the Self-Reporting Questionnaire (SRQ-20) for psychiatric disorders.

### ARTERIAL PRESSURE (AP) MEASUREMENT

AP was measured through the oscillometric method, using an internationally validated digital sphygmomanometer (Omron HEM-742). Three AP measurements were performed with 1-minute intervals. Data collection was performed after the in-person interview in order to ensure that the interviewee did not eat or smoke and remained seated, at rest, for at least 10 minutes before measurements.

### NUTRITIONAL STATUS ASSESSMENT

Nutritional status was assessed according to the body mass index (BMI), calculated by dividing the individual’s weight (in kg) by height squared (in m). Weight was measured by a previously calibrated portable digital scale with capacity for 150 kg and a precision of 50 g. The individual should stand on the center of the scale, barefoot and wearing light clothes. Stature was verified with a mobile stadiometer that reached measurements of up to 213 cm, with a precision of 0.35 cm. The individual should stand with the back touching the stadiometer, feet together, with an erect posture and looking forward, and the reading was done at the closest millimeter when the head-plate touched the individual’s head. For the BMI classification, we adopted the criteria established by the WHO and adapted by the National Institutes of Health (NIH): underweight (BMI < 18.50 kg/m^2^); normal weight (BMI ≥ 18.50 kg/m^2^ and ≤ 24.99 kg/m^2^); overweight (BMI ≥ 25.0 kg/m^2^ and ≤ 29.99 kg/m^2^); and obesity (BMI ≥ 30.00 kg/m^2^).^15^

### FAT PERCENTAGE ASSESSMENT

The assessment of body fat percentage was performed through an electric bioimpedance analysis with a 0.5% grading scale for body fat; this analysis assessed the lower segment of the body. The participant laid on a stretcher in the dorsal decubitus position, and electrodes were placed on predefined sites: the first electrode was placed directly below the joint of the middle finger; the second one was placed on the wrist joint; the third was placed between the second and third toes; and the fourth was placed between the ankle and the lower leg bone. The fat percentage was classified according to Lohman^16^ as: risk of diseases associated with malnutrition: ≤ 5% in men and ≤ 8% in women; below average: 6 to 14% in men and 9 to 22% in women; on average: 15% in men and 23% in women; above average: 16 a 24% in men and 24 to 31% in women; and risk of diseases associated with obesity: ≥ 25% in men and ≥ 32% in women.

### STUDIED VARIABLES

The dependent variable in this study was stress, constructed from an analysis of the literature on organizational stressors of psychosocial nature that cause adverse psychological reactions to workers. Based on principles by Paschoal & Tamayo,^17^ the outcome variable was created by combining two variables in this study: nervousness and tiredness. For constructing this proxy variable for stress, we used two questions: “Do you feel nervous, tense, or worried?” and “Do you get tired easily?” The answer options were “yes” or “no” for both questions; this way, individuals who answered “yes” to both questions were classified as stressed.

**Figure 1:**
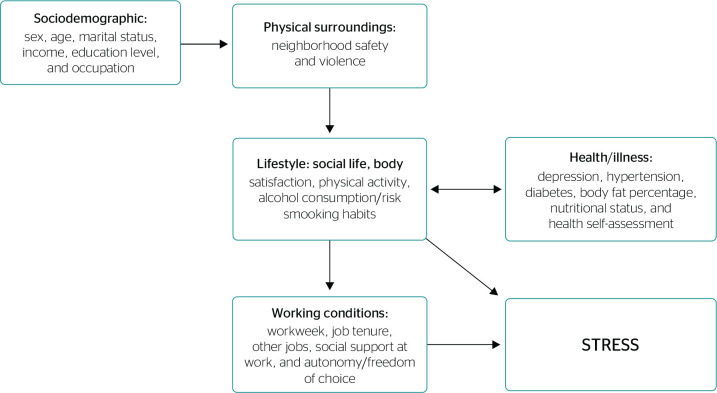
Conceptual hierarchical model for the analysis of variables associated with stress, Bahia, 2016.

A conceptual analysis model was built based on the literature for determinant factors of stress (Figure 1). The sociodemographic block was constructed using variables such as sex (male and female), age (22 to 39 years; 40 years or older), marital status (married/civil union; not married/single, divorced, or widowed), family income, education level (incomplete higher education: from the first year of primary education to incomplete higher education; higher education: from college education to post-doctoral education), and occupation (dichotomized between professor or technician/outsourced worker).

95%CI = 95% confidence interval.

Regarding the “physical surroundings” block, we assessed safety and violence perceived in the neighborhood, both with the following answer options: agree (completely or partially) and disagree (neither agree nor disagree, partially disagree, or completely disagree). The questions were extracted from the ELSA-Brasil neighborhood scale, which is a Likert-type scale.

As for the “lifestyle” block, we assessed “body satisfaction” (satisfied; dissatisfied, underweight; dissatisfied, overweight); for “physical activity (PA)”, we considered PA in its four domains (leisure, work, household, and commuting), and multiplied the weekly frequency of PA (in days) by the mean duration (in minutes) of walking performance or other moderate and vigorous PA. When considering vigorous PA, the duration was multiplied by two. Individuals who practiced 150 minutes or more of PA per week in at least one of the assessed domains were considered physically active.^10,14,18^ PA considered activities continuously performed for at least 10 uninterrupted minutes.^10^

Regarding lifestyle habits, alcohol consumption was calculated using the daily doses of alcohol reported by participants and risky alcohol use was considered when an individual consumed more than 30 g/day of alcohol (for men) or 15 g/day (for women). For smoking habits, two categories were considered according to the individuals’ answers: current smokes or does not currently smoke, being categorized as smokes/does not smoke.

For the “working conditions” block, we defined the following variables: job tenure (up to 2 years; more than 2 and less than 5 years; more than 5 and less than 8 years; or 8 years or more) and workweek (20 to 30 hours a week; 31 to 40 hours a week; or more than 40 hours a week).

The health/illness block consisted in fat percentage (below average/average; above average; or obesity), BMI, categorized into underweight/normal weight or overweight/obesity, and self-reported health status (very good/good, regular, or poor/very poor). Depression and diabetes were self-reported. As to hypertension, we used the mean value between the last two measurements, and individuals were considered hypertense when having a systolic arterial pressure ≥ 140 mmHg and/or a diastolic arterial pressure ≥ 90mmHg and/or using anti-hypertensive drugs.

### STATISTICAL ANALYSIS

We initially estimated the prevalence of stress among workers with a 95% confidence interval (95%CI). Independent variables were presented as absolute and relative frequencies. For verifying which factors were associated with stress, we performed a univariate analysis estimating PRs and calculating the respective CIs. A p-value ≤ 0.05 was considered significant. Data analysis was performed using Stata software, version 12.0.

### ETHICAL ASPECTS

This research proposal was approved by the Research Ethics Committee of the Multidisciplinary Institute on Health of Universidade Federal da Bahia (Certificate of Presentation for Ethical Appreciation No. 44496015 8 0000 5556).

## RESULTS

Out of 191 eligible individuals, 176 participated in this study. The population of this study comprised individuals employed by a higher education institution in the southeast region of BA. Among the observed losses, 7.85% happened due to refusal to participate in the longitudinal study.

Stress was present in 22.7% (95%CI: 16.48-28.98) of the population. Out of the 176 participants in this study, 71 (40.3%) were professors and 105 (59.7%) were technicians or outsourced employees (Table 1).

**Table 1:** Distribution of the population of workers according to sociodemographic characteristics, physical surroundings, lifestyle, working conditions, and health/illness, Bahia, 2016

Variables	n	Prevalence (%)	95%CI
Sociodemographic			
Sex			
Male	90	51.1	43.68-58.59
Female	86	48.9	41.41-56.32
Age (years)			
22-39	129	73.3	66.70-79.90
40 or older	47	26.7	20.10-33.31
Marital status			
Married	119	67.6	60.63-74.60
Not married	57	32.4	25.40-39.37
Family income (times the minimum wage)			
More than 5	100	57.1	49.74-64.55
3 to 5	24	13.7	8.57-18.86
Less than 3	51	29.1	22.34-35.94
Education level			
Higher education	122	69.3	62.44-76.20
Incomplete higher education	54	30.7	23.80-37.56
Occupation			
Professor	71	40.3	33.02-47.66
Technician/outsourced worker	105	59.7	52.34-66.98
Physical surroundings			
Safe neighborhood			
Agree	103	58.5	34.13-48.83
Disagree	73	41.5	51,17-65.87
Violent neighborhood			
Agree	87	49.4	41.97-56.90
Disagree	89	50.6	43.11-58.03
Lifestyle			
Body satisfaction			
Satisfied	26	14.8	9.48-20.07
Dissatisfied (underweight)	18	10.2	5.71-14.75
Dissatisfied (overweight)	132	75.0	68.54-81.46
Physical activity			
Physically active	139	79.4	73.38-85.48
Physically inactive	36	20.6	14.52-26.62
Alcohol consumption/risk			
No	123	70.3	63.45-77.12
Yes	52	29.7	22.88-36.55
Smoking habits			
Non-smoker	168	95.5	79.41-328.44
Smoker	8	4.6	25.39-168.34
Working conditions			
Job tenure (years)			
1 or less	44	25.0	18.54-31.46
2 to 5	47	26.7	20.10-33.31
5 to 8	43	24.4	18.02-30.84
More than 8	42	23.9	17.50-30.22
Workweek (hours)			
20 to 30	50	28.4	21.68-35.14
31 to 40	74	42.1	34.68-49.41
More than 40	52	29.6	22.74-36.35
Union membership			
Yes	71	40.3	33.02-47.66
No	105	59.7	52.34-66.98
Health/illness			
Depression			
No	148	84.1	78.63-89.54
Yes	28	15.9	10.45-21.37
Arterial hypertension			
No	134	76.1	69.78-82.50
Yes	42	23.9	17.50-30.22
Diabetes mellitus			
No	172	97.7	95.50-99.95
Yes	4	2.3	00.04-04.50
Body fat percentage			
Below average/on average	28	18.0	11.90-24.04
Above average	66	42.3	34.47-50.15
High	62	39.7	31.98-47,51
Nutritional status			
Underweight/normal weight	75	47.8	39.87-55.67
Overweight/obesity	82	52.2	44.32-60.13
Stress			
No	136	77.3	71.02-83.52
Yes	40	22.7	16.48-28.98
Health self-assessment			
Very good/good	116	65.9	58.84-72.98
Regular	52	29.6	22.74-36.35
Poor/very poor	8	4.6	01.44-07.65

Most (51.1%) participants were male, aged between 22 and 39 years (73.3%), married (67.6%), with a monthly family income (57.1%) of more than five times the minimum wage, and 69.3% had a higher education degree. As for the physical surroundings block, 58.5% of the participants agreed they had safety in their neighborhood; however, 49.4% stated they had suffered some type of violence in the neighborhood. (Table 1).

Regarding the lifestyle block, most of the population (75%) in this study revealed they were not satisfied with their own bodies. Among all participants, 79.4% were classified as physically active in at least one of the assessed domains of PA, 29.7% presented risky alcohol use, and 4.6% of them were smokers. Considering the working conditions block, 26.7% of the population had a job tenure of 2 to 5 years. Most of them (42.1%) had a workweek of 31 to 40 hours and did not have other sources of income (82.4%) (Table 1).

As for the health/illness block, 23.9% were classified as having arterial hypertension; self-reported diabetes and depression were observed in 2.3% and 15.9% of individuals, respectively. Regarding their body fat percentage, 42.3% were classified as above average and 52.2%, as overweight/obese. Among all individuals, 65.9% self-assessed their health as “very good/good” (Table 1).

Higher prevalence rates were observed for stress among women (34.9%) in relation to men, with statistical significance (p = 0.001); this was also observed in individuals aged between 22 and 39 years (24.8%) and in unmarried individuals (24.6%). However, no statistical significance was observed for these variables. Moreover, a higher prevalence of stress was observed in employees presenting a family income of more than five times the minimum wage (28%). We observed that the lower the family income was, the lower the prevalence of stress in this study; however, this aspect did not have statistical significance. Individuals with incomplete higher education and belonging to the technicians/outsourced employees group presented lower prevalence rates for stress: 13% and 15.2%, respectively - only the latter presented statistical significance (Table 2).

**Table 2:** Univariate analysis of variables that were present in the theoretical model with occupational stress in the population of workers of a higher education institution, Bahia, 2016

Variables	n	Prevalence (%)	Prevalence ratio	95%CI	p-value^*^
Sociodemographic					
Sex					0.001
Male	10	11.11	1.00	-	
Female	30	34.88	3.14	1.63-6.04	
Age (years)					0.292
22 to 39	32	24.81	1.00	-	
40 or older	8	17.02	0.69	0.34-1.38	
Marital status					0.687
Married	26	21.85	1.00	-	
Not married	14	24.56	0.89	0.50-1.57	
Family income (times the minimum wage)					0.169
More than 5	28	28.00	1.00	-	
3 to 5	5	20.83	0.74	0.32-1.73	
1 to 3	7	13.73	0.49	0.23-1.05	
Education level (years of schooling)					0.055
Higher education	33	27.05	1.00	-	
Incomplete higher education	7	12.96	2.09	0.98-4.43	
Occupation					
Professor	24	33.80	0.45	0.26-0.79	0.005
Technicians/outsourced employees	16	15.24	1.00	-	
Physical surroundings					
Safe neighborhood					0.215
Agree	20	19.42	1.00	-	
Disagree	20	27.40	0.71	0.41-1.22	
Violent neighborhood					0.660
Agree	21	24.14	1.13	0.65-1.95	
Disagree	19	21.35	1.00	-	
Lifestyle					
Body satisfaction					0.025
Satisfied	3	11.54	1.00	-	
Dissatisfied (underweight)	8	44.44	1.02	0.57-14.75	
Dissatisfied (overweight)	29	21.97	0.75	0.69-0.81	
Physical activity					0.028
Physically inactive	13	36.11	1.00	-	
Physically active	27	19.42	0.54	0.56-2.03	
Alcohol consumption/risk					0.467
No	30	24.39	1.00	-	
Yes	10	13.23	0.79	0.42-1.50	
Smoking habits					0.514
Non-smoker	39	23.21	1.00	-	
Smoker	1	12.50	0.51	0.69-3.28	
Workweek (hours)					0.673
20 to 30	10	20.00	1.00	-	
31 to 40	16	21.62	1.08	0.53-2.19	
More than 40	14	26.92	1.35	0.66-2.75	
Job tenure (years)					0.004
1 or less	7	15.91	1.00	-	
2 to 5	7	14.89	0.94	0.36-2.46	
5 to 8	8	18.6	1.17	0.46-2.95	
More than 8	18	42.86	2.69	1.25-5.79	
Union membership					0.294
No	21	20.00	1.00	-	
Yes	19	26.76	1.34	0.78-2.31	
Health/illness					
Depression					0.000
No	25	16.89	1.00	-	
Yes	15	53.57	3.17	1.93-5.21	
Arterial hypertension					0.158
No	34	25.37	1.00	-	
Yes	6	14.29	3.49	0.25-1.25	
Diabetes mellitus					0.000
No	37	21.51	1.00	-	
Yes	3	75.00	3.49	1.85-6.58	
Body fat percentage					0.509
Below average/on average	8	28.57	1.00	-	
Above average	18	27.27	0.95	0,47-1.94	
High	12	19.35	0.68	0.31-1.47	
Nutritional status					0.157
Underweight/normal weight	22	29.33	1.00	-	
Overweight/obesity	16	19.51	0.67	0.38-1.17	
Health self-assessment					0.000
Very good/good	23	19.83	1.00	-	
Regular	11	21.15	1.07	0.56-2.03	
Poor/very poor	6	75.00	3.78	2.20-6.52	

* p-value calculated through the chi-squared test.

95%CI = 95% confidence interval.

We observed a higher prevalence (44.4%) of stress among individuals who were dissatisfied with their bodies due to low weight, with statistical significance (p = 0.025). Physical inactivity was also associated with this outcome, that is, individuals who practiced less than 150 minutes of PA a week presented a higher prevalence of stress (36.1%), with statistical significance (p = 0.028). Individuals who displayed risky alcohol use and those who reported being smokers presented lower prevalence rates for stress (13.2% and 12.5%, respectively) when compared to the reference categories, but this finding did not have statistical significance (Table 2).

When it comes to working conditions, the prevalence rates observed for stress were higher among individuals who worked more than 40 hours a week (26.9%) and had a job tenure of 8 years or more (42.9%); the latter was statistically associated with the outcome (p = 0.004) (Table 2).

Higher prevalence rates of stress were observed among workers with depression (53.6%), diabetes (75%), and in individuals who assessed their own health as poor or very poor (75%), being positively and significantly associated with the outcome (p = 0.000) in individuals with a fat percentage below average/on average (28.6%), with a nutritional status of underweight/normal weight (29.3%), and who did not have hypertension (25.4%); these were not statistically associated with the outcome (Table 2).

After adjustment, the following remained positively and significantly associated with stress: occupation as a professor (PR = 2.11; 95%CI: 1.22-3.66), individuals diagnosed with depression (PR = 2.43; 95%CI: 1.42-4.13), and those who self-assessed their health as poor/very poor (PR = 4.79; 95%CI: 2.71-8.49). The female sex variable (PR = 1.90; 95%CI: 0.97-3.71), although without statistical significance, was maintained in the final model as an adjustment factor (Table 3).

**Table 3:** Final model after Poisson regression analysis, with stress as dependent variable

Variables	Adjusted PR (95%CI)	p-value^*^
Occupation		
Technicians/outsourced employees	1.00	
Professors	2.11 (1.22-3.66)	0.008
Depression		
No	1.00	
Yes	2.43 (1.42-4.13)	0.001
Health self-assessment		
Very good/good	1.00	
Regular	1.43 (0.78-2.62)	0.251
Poor/very poor	4.79 (2.71-8.49)	0.000

* Calculated by a Poisson regression.

95%CI = 95% confidence interval; PR = prevalence ratio.

## DISCUSSION

In this work, the variables who remained positively associated with stress were professorship as occupation, a diagnosis of depression, and a health self-assessment as poor/very poor.

The prevalence of stress (22.7%) in this work was lower than that found in a study performed among professors of a public institution in Teresina, in the state of Piauí, where 42.9% of the participants presented mild stress.^8^ The observed difference may have been due to the fact that this study had, as its target, not only professors, but also technicians and outsourced employees. In a study performed with 1,000 Brazilian workers in the cities of Porto Alegre and São Paulo, published by the International Stress Management Association (Brazil), 70% of the participants suffered with stress, and its largest motivation was related to the professional domain. This study encompassed various professions. Most of the interviewees stated that stress was associated with work, such as long hours and excess tasks.^19^

In this study, occupation was a factor that remained associated with stress. Individuals in the group of professors were considered more stressed than the group of technicians/outsourced employees. Teaching is one of the professions that are more prone to developing stress and other psychological disorders. This is due to the fact that this occupation is related with a high workload, activity organization, planning and overseeing classes, and a continuous search for improving one’s curriculum, in addition to extracurricular activity management, which generates a psychological environment.^8,9^

In addition, professors are responsible for helping students with their healthy progress, considering individual differences, and respecting their learning abilities. Therefore, matters related to the work characteristics also contribute to a high stress level. Factors such as interactions between the environment and organizational conditions, the content of work, and the tasks, efforts, and individual characteristics of workers can significantly interfere with occupational health, possibly being beneficial or damaging to health. When considering professors, one should consider fast decision-making, balancing demands, and relationships between colleagues, higher-ranking employees, and students.^20,21^

Although not statistically significant, a higher prevalence of stress was seen in the age group of individuals aged 22 to 39 years. According to the literature, professional experience and theoretical knowledge for facing challenges at work can provide greater confidence for decision-making and thus be allies against stress in older employees when compared to the younger ones.^11^

In this study, workers classified as physically active presented a lower prevalence of stress in the bivariate analysis. Although this did not remain associated in the multivariate analysis, PA has shown considerable efficacy in results related to work, such as absence of disease, higher satisfaction, lower occupational stress, increased productivity, among others.^22^ Moreover, recent studies state that the lack of PA can contribute to important physiological and psychological problems, such as increased obesity, cancer, metabolic syndrome, cardiovascular diseases, worse body satisfaction, and lower self-esteem. Instead of PA being used as a tool for coping with stress, individuals sometimes resort to unhealthy behaviors as a way of facing and expressing their emotions.^23^

Depression was a comorbidity that remained associated with the studied event. In agreement with our findings, Donato et al.^24^ reported that individuals diagnosed with depression presented high prevalence rates of stress. According to Meyer et al.,^25^ if a stressed individual does not reveal what he or she feels, this absence of demonstration of feelings and emotions slowly generates internal conflicts and, consequently, depression. Moreover, it can have a negative impact on quality of life, work satisfaction, and relationships with colleagues, causing more stress and worsening of the individual’s depressive state.^26^ Studies propose that mental health interventions such as cognitive-behavioral therapy, when performed at the workplace, could decrease the levels of depression symptoms among workers.^27,28^

Shen et al.^20^ state that professors are not only responsible for transmitting intellectual and moral knowledge to students, but they need to keep up with modern technological evolution, which requires the understanding of new learnings. Therefore, some professionals may feel unable to cope with the tension, resulting in exhaustion, which can contribute to the development of depression.^20^

The PR for stress was 3.79 times higher among those who self-assessed their health as poor/very poor in comparison with those who self-assessed their health as good/very good. Corroborating these findings, Reis et al.^29^ consider that factors affecting the psychological domain, such as stress, can influence the health perception of individuals.

The self-assessment of health is a measure that encompasses various aspects such as demographic, socioeconomic, cultural, and lifestyle factors, in addition to issues related to the work environment and health status. As to the work characteristics, it is known that the exposure to divergent, exhaustive, and stressful conditions is a risk factor for the psychological health of individuals.^30,31^

Considering our study design, this study has limitations referring to causal inferences. The population size may have prevented some associations from being observed.

## CONCLUSIONS

The prevalence of stress among workers of this educational institution was higher among professors, individuals diagnosed with depression, and those who self-assessed their health as poor/very poor.

We highlight that the lower prevalence of stress observed among workers does not eliminate the health risk in the population exposed to stressful situations. In the structural context of general working conditions, actions towards prevention and promotion of occupational health at the academic and institutional levels need to be established according to the needs of this specific population.

Therefore, the results of this study will serve as subsidy for the elaboration of policy interventions aimed at the occupational environment, considering that individuals are prone to stress in this scenario.
